# Potential Changes in Rat Spermatogenesis and Sperm Parameters after Inhalation of *Boswellia papyrifera* and *Boswellia carterii* Incense

**DOI:** 10.3390/ijerph10030830

**Published:** 2013-02-28

**Authors:** Mukhtar Ahmed, Nasser Al-Daghri, Majed S. Alokail, Tajamul Hussain

**Affiliations:** 1 Transmission Electron Microscope Unit, Research Centre, College of Science, King Saud University, P.O. Box 2455, Riyadh 11451, Saudi Arabia; 2 Biomarkers Research Program, Biochemistry Department, College of Science, King Saud University, Riyadh 11451, Saudi Arabia; E-Mails: aldaghri2011@gmail.com (N.A.-D.); malokail@ksu.edu.sa (M.S.A.); 3 Centre of Excellence in Biotechnology Research, King Saud University, Riyadh 11451, Saudi Arabia; E-Mail: tajamulh4@yahoo.com

**Keywords:** *B. Papyrifera*, *B. Carterii*, albino rats, spermatogenesis, sperm parameters

## Abstract

In this study the effect of *Boswellia papyrifera* (*B. papyrifera*) and *Boswellia carterii* (*B. carterii*) smoke exposure on spermatogenesis and sperm parameters in male albino rats was investigated. Rats (n = 11) were exposed daily in smoking chambers to smoke emanated by burning 4 g each of either *B. papyrifera* or *B. carterii* for 48 days. At the end of exposure duration rats were killed, and the testes were excised and analysed for histopathological and ultrastructural changes. Sperm analysis including total sperm count, motility, velocity and relative percentage of abnormal sperms were recorded. Rats exposed to *B. papyrifera and B. carterii* showed significant disturbances in spermatogenetic patterns and changes in sperm kinetics compared to unexposed rats. Atrophied seminiferous tubules with dynamic changes were also noticed. The boundaries of intercellular and intracellular vacuoles were seen in the Sertoli cells. Furthermore, in spermatids acrosomal vesicles were not fully formed. Degenerating spermatids were devoid of their nuclear membrane with electron dense matrix and vacuolization. Structural changes in Leydig cells were observed. Sperm analysis in exposed rats exhibited significant decrease in the sperm count, motility, speed and an increase in sperm anomalies when compare to controls. These findings demonstrate that the *B. papyrifera* and *B. carterii* smoke affects the process of spermatogenesis and sperm parameters and indicate the detrimental effects of these incense materials on human reproductive system.

## 1. Introduction

*Boswellia papyrifera* and *Boswellia carterii*, commonly known as Arabian incense, are traditionally used in the Ayurvedic system of medicine to treat arthritis. The chemical compositions of these plants were well characterized and reportedly include isoincensole and incensole acetate as the main diterpenic components [1,2]. *Boswellia* wood and its oil is used as an important ingredient in oriental perfumes and religious ceremonies in ancient Persia, Babylon, Greece and Rome. Studies also suggest that boswellic acid exerts significant anticancer, antimicrobial and immune-potent effects [3].

Apart from its therapeutic values, boswellic acid exposure has been shown to cause severe pulmonary changes and impair the lung function in rats [4,5]. In a recent study, the incense smoke exposure to rats significantly decreased the liver alkaline phosphatase (ALP), alanine aminotransferase (ALT) and aspartate aminotransferase (AST) glutathione (GSH) and the activities of superoxide dismutase (SOD), catalase (CAT), and glutathione peroxidase (GPx), and significantly increased the lipid peroxidation [6]. Long-term exposure to incense smoke is associated with weight loss, transiently increased plasma leptin, increased triglycerides, and decreased HDL-cholesterol, suggesting the adverse metabolic effects of incense smoke [7].

A number of studies have reported the pathological and pharmacological effect of incense smoke on lung and liver tissues. However, the information related to the effects of incense smoke exposure on spermatogenesis and sperm kinetics is limited. Therefore, we aimed to examine the potential toxic effects of incense exposure on rat spermatogenesis and sperm physiology.

## 2. Experimental Section

### 2.1. Animals and Incense

Male Wistar albino rats (*Rattus norvegicus*) aged 7–8 weeks, weighing 200–210 g were obtained from the Animal Care Center, College of Science, King Saud University; Riyadh, Saudi Arabia. The Ethics Committee of the Experimental Animal Care Center approved the study. Animals were housed in a temperature-controlled room on a 12-h light/dark cycle and had access to water and normal chow diet *ad libitum*. *B. papyrifera* (crude incense) and *B. carterii* (refined incense) were obtained locally.

### 2.2. Exposure to *B. papyrifera* and *B. carterii* Smoke

After one week acclimatization period, rats were randomly divided into three groups, *viz*. groups 1, 2 and 3 with each group consisting of 11 animals. Each group of rats was housed separately from the other groups to avoid the cross exposure to incense smoke. Rats in group 1 served as control and were kept in normal fresh air, while rats in group 2 and 3 were exposed to *B. papyrifera* and *B. carterii* smoke, respectively, in a smoking chamber as described by Wang *et al.* [8]. The rats were exposed daily to the smoke emanating from the burning of 4 g of each incense material for 4 months. Smoke exposure durations lasted for 30–40 min/day. The dose and duration of incense exposure followed in this study was based on the optimized conditions from our previous studies [6,7]. At the end of exposure duration, animals were killed by cervical dislocation.

### 2.3. Sperm Analysis

The cauda epididymis and seminal vesicle excised from the rats were minced into phosphate buffered glucose saline (PBGS); and a clear suspension devoid of debris was obtained. Epididymal plasma was used for the analysis of total sperm count, sperm motility, forward velocity and relative percentage of abnormal sperms. The total sperm count and motility were calculated according to the method of Besley *et al.* [9] using Neubauer’s haemocytometer. The spermatozoa were allowed to settle down in haemocytometer for total sperm count by keeping them in a humid chamber (4 °C) for one hour. The sperm count was recorded in R.B.C counting chambers [10]. Similarly, total numbers of motile sperm were calculated immediately using phosphate buffer saline instead of spermicidal solution and the time gap between counts were 0.5 to 10 s. The forward velocity of sperm was calculated according to Ratnasoorya [11]. The assessment was made under light microscope, fitted with a movable mechanical stage and a calibrated ocular micrometer, at 400× magnification. A drop of sperm suspension was transferred to a clean glass slide and the initial place and time of each sperm was recorded. The time taken for forward movement of sperm from the initial place within the microscopic field was recorded using a stopwatch. The procedure was repeated for 10 spermatozoa for each sample and the average forward velocity of sperm was calculated and expressed as µm/sec. The relative proportions of abnormal sperms were analyzed by Bauer *et al*. [12]. Briefly, equal volumes of cauda epididymal plasma and 5% NaHCO_3_ were placed in a centrifuge tube, mixed well and centrifuged for 5 min at 4,000 rpm. The supernatant was discarded and 5 mL of normal saline was added to the precipitate, mixed well and centrifuged again. The procedure was repeated two to three times until a clear precipitate was obtained. To the final precipitate, a few drops of normal saline were added, mixed thoroughly and a smear was prepared on a clean slide. The smear was dried at room temperature, fixed by heating over the flame for two to three seconds. Then the smear was flushed with 95% alcohol, drained and dried. Smear was stained in Ziehl Neelson’s Carbol Fuchsin diluted with equal volume of 95% alcohol for 3 min and counter stained with 1:3 (v/v) aqueous solution of Loeffer’s methylene blue for 2 min. After staining, the smear was rinsed in water and dried in air. The abnormal sperms were characterized based on the presence of double tails, detached heads, detached tails, mid piece bending and irregular heads. The relative proportions of normal and abnormal sperm were expressed in terms of percentages. Fructose levels of epididymal fluid and seminal plasma were measured by Bauer *et al*. [12]; briefly, 0.1 mL of epididymal fluid and seminal plasma mix was diluted with 2.9 mL distilled water and the mixture was added to 0.5 mL 0.3 N barium hydroxide (95 mg barium hydroxide in 2 liters of distilled water). Contents were mixed and added to 0.5 mL 5.0% (0.175 M) zinc sulfate solution, mixed well, allowed to stand for few minutes and centrifuged at 5,000 rpm for 10 min. Supernatant was transferred to a fresh tube and 2 mL of it was mixed with 2 mL of 0.1% resorcinol solution and then with 6 mL of 10N HCL, mixed well, heated at 90 °C for 10 min and cooled to room temperature. Two mL of 2% working standard, equivalent to 200 mg/100 mL fructose in the seminal fluid was similarly processed. Absorbance of test and standard samples was measured at 490 nm. The average fructose content of fluid was calculated and expressed as mg fructose/100 mL (absorbance of samples**/**absorbance of standard ×200).

### 2.4. Histopathological and Ultrastructural Study

The testes were harvested from the rats and fixed in 10% formosaline. Paraffin sections (4–5 μm) were prepared and stained with hematoxylin and eosin and examined under light microscopy for histological changes. Ten sections were randomly selected and 10 different view fields per section were counted. For Transmission Electron Microscope (TEM) studies, tissues were sliced into 1 mm^3^ slices and fixed in 3% buffered glutaraldehyde (sodium cacodylate buffer, pH 7.2) for 4 h at 4 °C. Tissue specimens were then post-fixed in 1% osmium tetaroxide (OsO_4_) in cacodylate buffer (pH 7.2) for 2 h at 4 °C. Dehydration of the fixed tissues was performed using ascending grades of ethanol. Tissue slides were transferred to epoxy resin via propylene oxide. After impregnation with the pure resin (SPI Resin), tissue specimens were embedded in the same resin mixture [13]. Semi-thin sections (300 nm thickness) were prepared for the purpose of tissue orientation and stained with toluidine blue. Ultrathin sections of silver shades (65–70 nm) were cut using an ultra-microtome (Leica Ultracut UCT, Tokyo, Japan) with a diamond knife and stained with uranyl acetate and lead citrate. Stained sections were finally observed under TEM (JEOL; JEM 1011 Tokyo, Japan) operating at 80 KV.

### 2.5. Statistical Analysis

Data were expressed as mean values ± SE. Student’s *t*-test was applied to compare the significance of difference. Differences were considered statistically significant when *p* < 0.001.

## 3. Results

### 3.1. Sperm Analysis

Sperm parameters including total sperm count, total number of motile sperm (usually motile sperm swim forward in an essentially straight line, whereas non-motile sperm swim, but abnormal paths, such as in circles), forward velocity and percentage of abnormal sperm, fructose content of epididymal and seminal plasma were compared between exposed and unexposed control rats. Relative to control group, animals exposed to *B. papyrifera* and *B. carterii* showed a significant decrease in the total sperm count, the total number of motile sperm and the forward velocity of the sperm (Table 1, *p* < 0.001). The percentages of abnormal sperm were increased (*p* < 0.001); whereas the fructose levels were significantly decreased (3-fold, *p* < 0.001).

### 3.2. Histology and Morphometric Study

**Figure 1:**
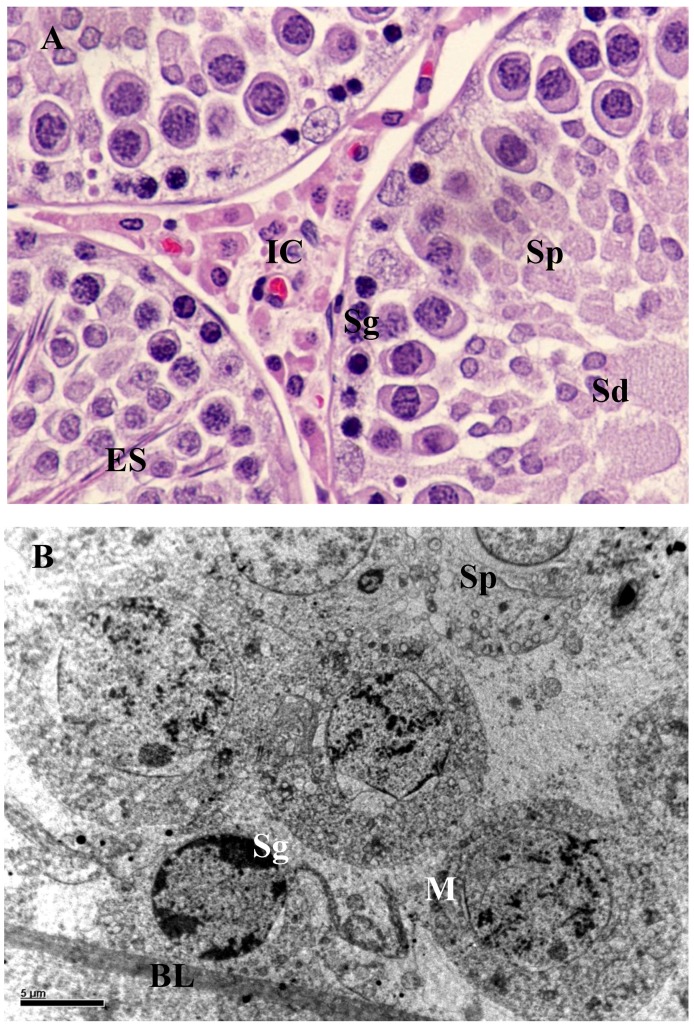
(**A**) Section of the seminiferous tubules of control rat showing normal spermatogenesis with normal features of spermatogonia (Sg), spermatocytes (Sp), spermatids (Sd), elongated spermatids (ES) and interstitial cells (IC) × 1,000. (**B**) Spermatogonia (Sg) rest upon the basal lamina (BL). Both spermatogonia (Sg) and spermatocytes (Sp) appear normal. Mitochondria (M) of germ cells exhibited in abundance with sign of degenerations 8,000×.

**Figure 2:**
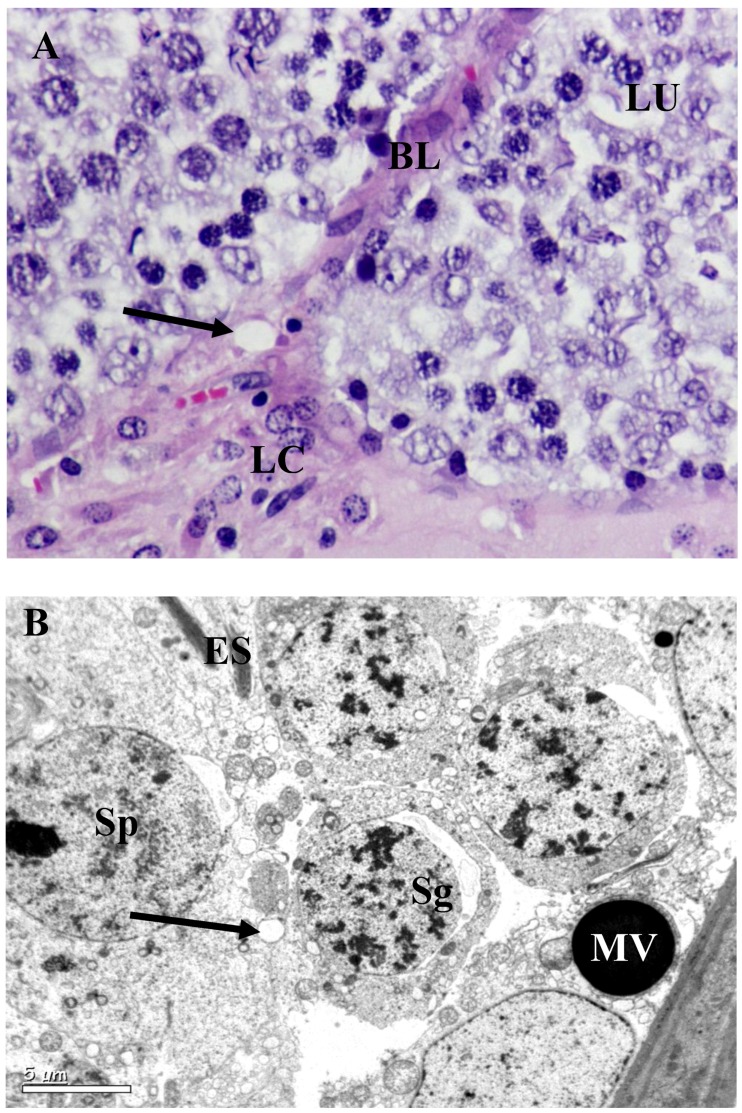
Testis of *B. papyrifera* smoke exposed rat. (**A**) Seminiferous tubules of the rat exposed to *B. papyrifera* exhibiting extensive damage on tubules and reduction in size. Disruption of seminiferous epithelium was evident. Spermatogenesis stopped at the primary spermatocytes stage. Interstitial spaces increased and atrophy of Leydig cells (LC); which are sparsely distributed. The germ cells showed overall decrease in cytoplasmic ground substance followed by vacuolization (arrow) at the basal lamina (BL) and towards the lumen (LU) × 1,000. (**B**) There was a complete disturbance in the spermatogonia (Sg) and spermatids (Sp). Disturbed elongated spermatids were seen at one corner (ES). Lysosome (arrow) like bodies was evident in the section. Vacuolization throughout the cytoplasm, cell debris and disturbed cell organelles were evident. A large micro vesicle (MV) was seen at the basal lamina 8,000×.

**Figure 3:**
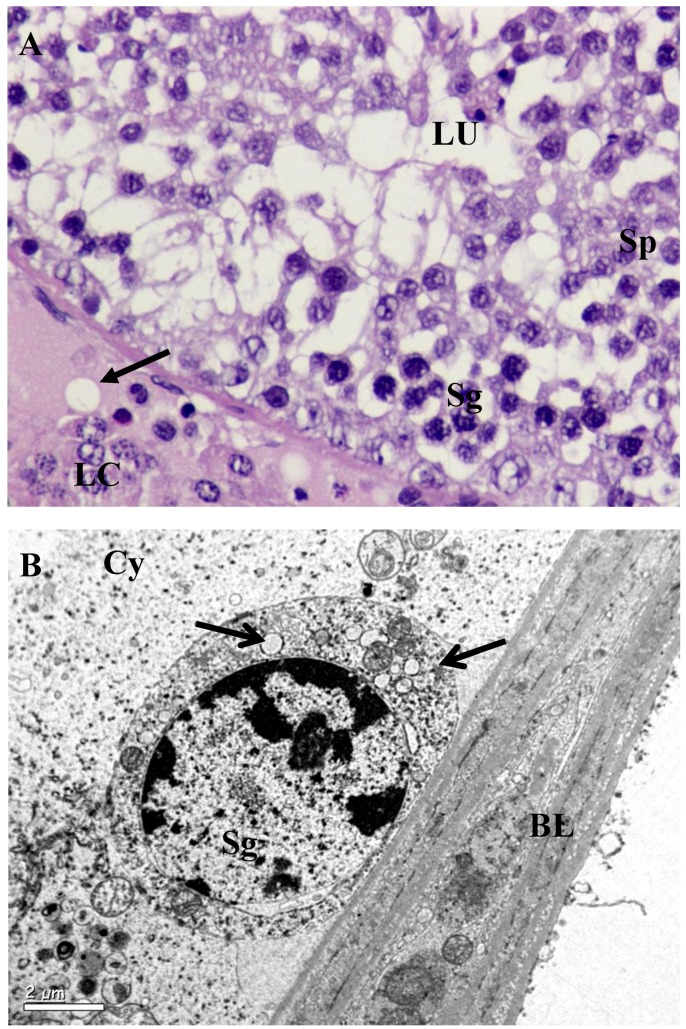
Testis of *B. carterii* smoke exposed rat. (**A**) Seminiferous tubules exposed to *B. carterii* showed severe effects on the tubules and overall reduction in their size. Degeneration of basal lamina was evident without basal cells. Spermatogenesis stopped completely at the primary spermatocyte stages as seen in the lumen (LU). Interstitial spaces increased and atrophy of Leydig cells were formed (LC). Germ cell such as Spermatogonia (Sg) and Spermatocytes (Sp) were completely disturbed. Cells showed overall decrease in cytoplasmic ground substance followed by vacuolization (arrows) × 1,000. (**B**) Vacuolization (arrow) in the spermatogonia was evident, which rested on the basal lamina (BL). The basal lamina appeared normal. Cytoplasm (Cy) hypertrophied with other organelles × 8,000.

The testis of control rats (Figure 1(A); Table 2) exhibited different stages in seminiferous elements comprising germ cells, Sertoli cells and interstitial cells, which were normal in appearance. Towards the lumen, the primary spermatocytes; secondary spermatocytes; early spermatids and elongated spermatids were associated with Sertoli cells. Mature spermatozoa were also visible in the same region. The rats exposed to *B. papyrifera* or *B. carterii* smoke showed atrophic tubules, and disturbance in the various stages of spermatogenesis. The basement membrane and tunica propria became thin and disrupted. Spermatogenesis was arrested either at the primary spermatocyte or at the spermatogonial stages. The Sertoli cells showed vacuolization and cell debris due to cytolysis. The intercellular spacing became wider, Leydig cells reduced in numbers and the interstitium contained mostly fibroblasts (Figure 2(A), Figure 3(A)). There was a significant increase in the seminiferous tubules per microscopic field as shown in Table 2 (*p* < 0.001). The diameter of seminiferous tubules decreased significantly and there was significant decrease in the total count of spermatogonia, spermatocytes, spermatids, Leydig cells, and Sertoli cells (Table 2, *p* < 0.001). A significant decrease (50–60 %) in the cells and nuclear diameters of spermatogonia, spermatocytes, spermatids and Leydig cells was also noticed (Table 3, Table 4, *p* ≤ 0.001).

### 3.3. Ultrastructural Changes in Testis

Testis of the unexposed control rats showed normal seminiferous cycles with different phases of development starting from spermatogonia to spermatids. Sertoli cell cytoplasm extended from the basal lamina of the seminiferous tubule to the lumen and surrounded the germ cells (Figure 1(B)). Dark spermatogonia and spermatocytes with a round nucleus and patchy chromatin materials were present adjacent to the basal lamina and connected to Sertoli cells by intercellular bridges. A large number of Golgi apparatus; granular and agranular endoplasmic reticulum were randomly seen in these germ cells. The different types of spermatids showed normal structure with well-defined nucleus, manchette with microtubules, acrosomic vesicles, Golgi complex and acrosomal granules (Figure 1(B)).

Leydig cells from control animals also showed normal structure in the interstitium and contained darkly stained compact cytoplasm with heterochromatin nuclei. Other cell types in the interstitium, such as lymphocytes, endothelial cells, smooth muscle cells and myoid cells appeared normal (Figure 1B). All the cyclic stages of spermatogenesis were seen with their regular shapes and sizes and formation of regular spermatozoa. In contrast, in rats exposed to *B. papyrifera* and *B. carterii*, the seminiferous tubules showed vacuolization and large intercellular spaces. At the region of basal lamina, spermatogonia and Sertoli cell nuclei lie normal. Intraepithelial vacuoles were found to consist of intercellular spaces and intracellular vacuoles in the cytoplasm of the Sertoli cells.

**Table 1:** Effect *B. papyrifera* and *B. carterii* smoke exposure on various sperm parameters and fructose content in cauda epididymal and seminal plasma of albino rats.

Group	Sperm parameters				Plasma fructose (mg/100 mL)	
	Total number of sperm (Total No. × 10^4^/mL)	Total number of motile sperm (Total No. × 10^4^/mL)	Velocity (µm/s)	Abnormal (%)	Cauda Epididymis	Seminal Vesicle
**I**	67.49 ± 1.29	55.39 ± 2.17	121.56 ± 1.70	11.80 ± 1.21	80.72 ± 2.13	110.58 ± 1.63
**Control**						
**II**	37.23 ± 1.31 ***	33.10 ± 1.16 ***	65.48 ± 1.51 ***	55.63 ± 1.47 ***	39.15 ± 1.91 ***	47.59 ± 1.37 ***
***B. papyrifera***						
**III**	31.47 ± 1.11 ***	27.70 ± 1.31 ***	61.86 ± 1.29 ***	59.58 ± 0.34 ***	29.64 ± 0.32 ***	43.71 ± 1.67 ***
***B.* carterii**						

*** *p* < 0.001.

**Table 2:** Effect *B. carterii* and *B. papyrifera* smoke exposure on total count of seminiferous tubules, germ cells, Leydig cells and Sertoli cells in the testis of albino rats.

Group	Number of					
	Seminiferous tubules in microscopic field (10×)	Spermatogonia	Spermatocytes	Spermatids	Leydig cells	Sertoli cells
**I**	15.50 ± 0.21	130.70 ± 2.39	549.55 ± 29.34	986.35 ± 7.28	57.30 ± 0.41	29.63 ± 1.56
**Control**						
**II**	21.13 ± 0.41 ***	90.50 ± 1.36 ***	427.31 ± 1.41 ***	762.30 ± 1.43 ***	27.12 ± 1.35 ***	20.81 ± 1.67 ***
***B. papyrifera***						
**III**	23.18 ± 0.40 ***	70.09 ± 2.36 ***	321.57 ± 4.21 ***	593.17 ± 3.3 ***	25.17 ± 1.77 ***	17.91 ± 1.39 ***
***B. carterii***						

*** *p* < 0.001.

**Table 3:** Effect *B. carterii* and *B. papyrifera* smoke exposure on diameter of seminiferous tubules, germ cells (µm) in the testis of albino rats.

Group	10×	100×		
	Seminiferous tubules (µm)	Spermatogonia (µm)	Spermatocytes (µm)	Spermatids (µm)
**I**	273.51 ± 1.27	11.18 ± 0.24	9.33 ± 0.31	9.00 ± 0.23
**Control**				
**II**	239.19 ± 5.43 ***	7.21 ± 0.63 ***	6.00 ± 0.27 ***	5.90 ± 0.33 ***
***B. papyrifera***				
**III**	261.16 ± 2.73 ***	5.21 ± 0.39 ***	5.39 ± 0.21 ***	4.56 ± 0.39 ***
***B. carterii***				

*** *p* < 0.001.

**Table 4:** Effect *B. carterii* and *B. papyrifera* smoke exposure on nuclear diameter (µm) of the germ cells in testis of albino rats.

Group	100× (µm)			
	Spermatogonia	Spermatocytes	Spermatids	Leydig cells
**I**	10.38 ± 0.19	8.45 ± 0.22	7.08 ± 0.20	9.74 ± 0.27
**Control**				
**II**	5.09 ± 0.31 ***	5.63 ± 0.35 ***	4.26 ± 0.17 ***	5.61 ± 0.21 ***
***B. papyrifera***				
**III**	4.34 ± 0.27 ***	4.28 ± 0.21 ***	3.83 ± 0.11 ***	4.00 ± 0.19 ***
***B. carterii***				

*** *p* < 0.001.

Degeneration started from cytoplasm of early stage of spermatids towards its nucleus and from acrosomal granule or Golgi complex. There was a great degree of degeneration in all the stages of spermatogenic cycle (Figure 2(B), Figure 3(B)). Bridges between Sertoli cells spermatids were disturbed and most mitochondrial cytoplasms were either disturbed or hypertrophied. Membrane bound proacrosome granules appeared in the Sertoli cell cytoplasm. In the acrosomic phase of spermatids, acrosomal vesicles showed disruption in the middle portion of the manchette and mitochondria in the cytoplasm exhibited ballooning or hypertrophy characteristics (Figure 2(B), Figure 3(B)). Other cell organelles in the cytoplasm were absent. Degenerating spermatids were totally devoid of their nuclear membrane with electron dense matrix and commencement of vacuolization (Figure 2(B), Figure 3(B)). In Leydig cells, (Figure 2(B), Figure 3(B)) the cytoplasmic inclusions appeared diminished and were vacuolated, with marked decrease in organelle content. The nuclei were less chromatic; mitochondria in the cytoplasm were swollen contributing to vacuolization. Pinocytotic vesicles on periphery of the cells were clearly visible. Lipid droplets and lysosomes were seen scattered. Other interstitial cells showed different types of fibroblasts cells.

## 4. Discussion

### 4.1. Sperm Analysis

In this study we evaluated the toxicity of *B. papyrifera* and *B. carterii* smoke exposure on the reproductive system in male Wistar rats. We found a significant increase in sperm anomalies with decreased sperm count, motility, sperm speed and the decreased fructose contents. The epididymis plays an important role in sperm development and sperm maturation, where it depends on the luminal environment of the epididymis; including its specific proteins. Extracts of plants like *Ocimum sanctum* leaves (*O. ocimum*) [10] and *Aegle marmelos* [14] have been reported to possess toxic effects on sperm parameters in rodent models. These findings are consistent with our study. It was suggested that these plant extracts cause androgen depletion at the target levels, particularly in the cauda epididymis thereby affecting physiological maturation of sperms [10]. Present observations of increased abnormal sperms, reduced sperm count, motility and sperm speed with *B. papyrifera* and *B. carterii* suggested that sperm anomalies in rats might have resulted from the alteration in the epididymal milieu due to androgen deficiency and/or due to toxic effects on cellular levels. Fructose has been reported to be a source of energy for the motility of the gametes [15]. Patel *et al.* demonstrated a positive correlation between seminal fructose and percentage of motile sperms [16]. In this study abnormal sperm motility was directly correlated to decreased levels of fructose in seminal plasma and epididymal fluid.

The oxidation of lipids was a crucial step in the pathogenesis of several diseases. Lipid peroxidation is a process generated naturally in small amounts in the body, mainly by the effect of several reactive oxygen species (hydroxyl radical, hydrogen peroxide, *etc.*) or by the action of several phagocytes. Since lipid peroxidation is a self-propagating chain-reaction, the initial oxidation of only a few lipid molecules can lead to significant tissue damage. Despite extensive research in the field of lipid peroxidation it has not yet been precisely determined if it is the cause or an effect of several pathological conditions. Lipid peroxidation has been implicated in diseases such as atherosclerosis, IBD, ROP, BPD, asthma, Parkinson’s disease, kidney damage, preeclampsia and others [17]. In this study, increased lipid peroxidation was correlated to damage in spermatozoa and testicular dysfunctions. Although cigarette smoke exposure to rats showed secretory dysfunction of the Leydig cells, deficiency in sperm maturation and spermatogenesis and significant reductions in epididymal sperm content, motility and infertility *in vivo* and *in vitro* [18,19], such effects have not been verified for incense smoke exposure.

### 4.2. Histology of Testis

In this study, the toxicity of *B. papyrifera* and *B. carterii* was evident by the arrest of spermatogenesis. It has been reported that reduced testicular weight and maturational arrest of the primary spermatocytes manifests androgen deficiency. The morphometric analysis confirms the adverse effect on the spermatocytes, spermatids and Leydig cells. These views strongly support our findings, since these stages were completely androgen dependent [20]. The adverse effects of *B. papyrifera* and *B. carterii* on the rat testis including tubular atrophy, abnormal appearance of seminiferous epithelium and Leydig cells were due to curtailing of androgen supply within the testis or it may be a direct effect on target tissues. Similar observations were reported of other plants such as *Azadirachta indica* [21], *Aegle marmelos* [14] and *O. sanctum* [22].

### 4.3. Ultrastructure of Testis

Spermatogenesis is a complex process in which germ cells supported by Sertoli cells undergo mitotic and meiotic divisions to produce elongated spermatids. Androgens produced by the interstitium cells play an important role in maintenance of spermatogenesis in all animals. A few studies have been already published so for to relate experimentally induced morphological changes in germ cells, Sertoli cells and Leydig cells in laboratory animals to their functions in regulating spermatogenesis [20]. Testosterone has shown to be essential for spermatogenesis, because it stimulates the conversion of round spermatids into elongated spermatids of the spermatogenetic cycle. Androgen deficiency disturbs spermiation process by altering spermatid-Sertoli cell junctions; which results in premature detachment of round spermatids from Sertoli cells and seminal epithelium [20]. Decreased testosterone levels have been associated with alterations in Sertoli and Leydig cells [23]. Treatment with different parts of the plants such as leaf powder of *Azadirachta indica* [24,25] crude garlic [23] and benzene extract of *O. sanctum* leaves [22] on ultrastructure of the rat testis revealed several changes and it can be summaries in three categories such as: (i) vacuolization in the Sertoli cells and germ cells; (ii) degeneration of mitochondria followed by vacuolization in spermatocytes and spermatids; and (iii) a decrease in nuclear density and ruptures of plasmatic membranes. Studies from Aladakatti and Nazeer Ahamed [22,24] and Alladakatti *et al.* [25] have shown that *Azadirachta indica* leaf powder and benzene extract of *Ocimum sanctum* leaves cause the disruption of intercellular bridges between germ cell-germ cells, germ cells-Sertoli cells or Sertoli cells-Sertoli cells in rats due to their antiandrogenic properties. In view of the dynamic role of androgen in the initiation and maintenance of spermatids, it is believed that the degenerative changes observed in the spermatids may be due to deprivation of androgens. [12,20] In this study *B. papyrifera* and *B. carterii* caused disruption of intercellular bridges between germ cell-germ cells or germ cells-Sertoli cells, probably due to toxicity of these plants at the tissue level. It is known that the function of bridge partitioning complexes has yet not been established. Collectively, the data demonstrated that bridges are not static structures, but are modified at specific phases of development, especially during spermiogenesis. The morphological changes observed do not provide definitive information on bridge function, but their initial description serves as a basis for the companion study, which specifically address the function of certain bridge components [26].

It is well known that Sertoli cells interact directly with germ cells and perform a number of functions critical to spermatogenesis, including compartmentalization of the seminiferous tubules, physical and metabolic support of germ cells, a secretion of numerous factors that promote germ cell viability and differentiation [24]. Proteins of Sertoli cells, mainly the androgen binding protein (ABP) are required to achieve a specific step in germ cell maturation. The concurrent appearance of numerous vacuoles in this study represents a morphological indicator of Sertoli cell damage. This idea has been supported by the results of *Azadirachta indica* [21] and *Ocimum sanctum* [22] treatments.

In this study the potential toxicity of *B. papyrifera* and *B. carterii* resulted in vacuolization of Sertoli cell cytoplasm and loss of cytoplasmic organelles and suggested the loss of metabolic activities. Thus degeneration and arrest of germ cells could be attributed to Sertoli cell factors responsible for germ cell maturation [27]. Thus, it may be suggested that the toxicity of *B. papyrifera* and *B. carterii* probably affects the Sertoli cells directly or via the blood stream. This view was strengthened by our previous findings on various tissues such as lungs, livers, and blood serum parameters [6,7].

## 5. Conclusions

Sperm anomalies and histopathological changes found in this study demonstrate that the *B. papyrifera* and *B. carterii* smoke exposure affects the process of spermatogenesis in rats. These findings indicate the detrimental effects of incense smoke exposure and may have relevance to humans constantly exposed to indoor incense smoke. Further studies are warranted to understand the molecular mechanisms in target organs.

## References

[1] Kulkani R.R., Patki P.S., Jog V.P., Gandage S.G., Patwardhan B. (1991). Treatment of osteoarthritis with a herbomineral formulation. A double-blind, placebo-controlled, cross-over study. J. Ethnopharmacol..

[2] Camarda L., Dayton T., Di Stefano V., Pitonzo R., Schillaci D. (2007). Chemical composition and antimicrobial activity of some oleogum resin essential oils from *Boswellia* spp. (Burseraceae). Ann. Chim..

[3] Huan M.T., Badmaev V., Ding Y., Liu Y., Xie J.G., Ho C.T. (2000). Anti-tumor and anti-carcinogenic effects of triterpenoid, beta-boswellic-acid. Biofactors.

[4] Al-Arafi S.A., Mubarak M., Alokail M.S. (2004). Ultrastructure of the pulmonary alveolar cells of rats exposed to Arabian mix incense (Ma’ amoul). J Biol Sci..

[5] Alokail M.S., Alarifi S.A. (2004). Histological changes in the lung of Wistar albino rats (*Rattus norvegicus*) after exposure to Arabian incense (Genus *Boswellia*). Ann. Saudi. med..

[6] Alokail M.S., Mohammad A.I., Al-Arafi S.A. (2011). Antioxidant enzyme activity and lipid peroxidation in liver of wistar rats exposed to Arabian incense. Animal. Bio. J..

[7] Alokail M.S., Al-Daghri N.M., Al-Arafi S.A., Draz H.M., Tajamul H., Yakout S.M. (2011). Long-term exposure to incense smoke alters metabolism in Wistar albino rats. Cell Bio. Fun..

[8] Wang X.D., Liu C., Bronson R.T., Smith D.E., Krinsky N.I., Russell M.L. (1999). Retinoid signaling and activator protein-1 expression in ferrets given beta-carotene supplements and exposed to tobacco smoke. J. Nat. Can. Inst..

[9] Besley M.A., Eliarson R., Gallegosm A.J., Moghissi K.S., Paulsen C.A., Prasad M.R.N. (1980). Laboratory Manual for the Examination of Human Semen and Semen Cervical Mucus Interaction.

[10] Mukhtar A., Nazeer A.R., Ravindranath H.A., Mukhtar A.M.G. (2011). Effect of benzene extract of *Ocimum sanctum* leaves on cauda epididymal spermatozoa of rats. Iranian J. Repro. Med..

[11] Ratnasooriya W.D. (1984). Effect of Atropine on fertility of female rat and sperm motility. Indian J. Exp. Boil..

[12] Bauer J.D., Ackermen P.G., Toro G. (1974). Clinical Laboratory Methods.

[13] Reyenolds E.S. (1963). The use of lead citrate at high pH as an electron opaque stain in electron microscopy. J. Cell Biol..

[14] Chauhan A., Agarwal M. (2008). Reversible changes in the antifertility induced by *Aegle marmelos* in male albino rats. Sys. Biol. Repro. Med..

[15] Mann T., Nam T. (1964). Fructose, Polyols, and Organic Acids. The Biochemistry of Semen and of the Male Reproductive Tract.

[16] Patel S.M., Skandhan K.P., Mehta Y.B. Seminal plasma fructose and glucose in normal and pathological conditions. Acta Eur. Fertil..

[17] Mylonas C., Kouretas D. (1999). Lipid peroxidation and tissue damage. In Vivo.

[18] Yamamoto Y., Isoyama E., Sofikitis N., Miyagawa I. (1998). Effects of smoking on testicular function and fertilizing potential in rats. Urol. Res..

[19] Kapawa A., Giannakis D., Tsoukanelis K., Kanakas N., Baltogiannis D., Agapitos E., Loutradis D., Miyagawa I., Sofikitis N. (2004). Effects of paternal cigarette smoking on testicular function, sperm fertilizing capacity, embryonic development, and blastocyst capacity for implantation in rats. Andrologia.

[20] Beardsley A., O’Donnell L. (2003). Characterization of normal spermiation and spermiation failure induced by hormone suppression in adult rats. Biol. Repro..

[21] Aladakatti R.H., Nazeer A.R. (2006). *Azadirachta indica* A. Juss induced changes in spermatogenic pattern in albino rats. J. Nat. Remd..

[22] Aladakatti R.H., Mukhtar A., Nazeer A.R., Ghodesawar M.G. (2010). Effect of benzene leaf extract of *Ocimum sanctum* on testis and spermatogenic pattern in albino rats. Int. J. Curr. Res..

[23] Yang Z.W., Kong L.S., Guo Y., Yin J.Q., Mills N. (2006). Histological changes of the testis and epididymis in adult rats as a result of Leydig cell destruction after ethane dimethane sulfonate treatment: A morphometric study. Asian J. Androl..

[24] Aladakatti R.H., Nazeer A.R. (2005). Changes in Sertoli cells induced by *Azadirachta indica* A. Juss leaves in albino rats. J. B. Clin. Physiol. Pharmacol..

[25] Aladakatti R.H., Nazeer A.R. (2005). Ultrastructural changes in Leydig cell and cauda epididymal spermatozoa induced by *Azadirachta indica* leaves in albino rats. Phyto. Res..

[26] Russell L.D., Griswold M.D. (1993). The Sertoli Cell. Clearwater.

[27] Lohiya N.K., Mishra P.K., Pathak N., Manivannan B., Bhande S.S., Panneerdoss S., Sriram S. (2005). Efficacy trial on the purified compounds of the seeds of *Carica papaya* for male contraception in albino rat. Reprod. Toxicol..

